# Changes in transfusion and fluid therapy practices in severely injured children: an analysis of 5118 children from the TraumaRegister DGU®

**DOI:** 10.1007/s00068-020-01423-z

**Published:** 2020-06-29

**Authors:** Florian Piekarski, Jost Kaufmann, Thomas Engelhardt, Florian J. Raimann, Thomas Lustenberger, Ingo Marzi, Rolf Lefering, Kai Zacharowski, Patrick Meybohm, TraumaRegister DGU

**Affiliations:** 1 Department of Anaesthesiology, Intensive Care Medicine and Pain Therapy, University Hospital Frankfurt, Goethe University, Frankfurt, Germany; 2 Department for Paediatric Anaesthesia, Children’s Hospital Cologne, Cologne, Germany; 3 Faculty of Health, University of Witten/Herdecke, Witten, Germany; 4 Department for Anesthesia, Montreal Children’s Hospital, Montreal, Canada; 5 Department of Trauma, Hand and Reconstructive Surgery, University Hospital Frankfurt, Goethe-University, Frankfurt, Germany; 6 IFOM, Institute for Research in Operative Medicine, Faculty of Health, University Witten/Herdecke, Cologne, Germany; 7 Department of Anaesthesia and Critical Care, University Hospital Würzburg, Würzburg, Germany; 8 Committee On Emergency Medicine, Intensive Care and Trauma Management (Sektion NIS) of the German Trauma Society (DGU), Berlin, Germany

**Keywords:** Paediatric trauma patients, Transfusion practice, Patient blood management, Outcome, Mortality, Fluid therapy, Volume therapy, Serious injured children, TraumaRegister DGU® (TR-DGU)

## Abstract

**Purpose:**

Trauma is the leading cause of death in children. In adults, blood transfusion and fluid resuscitation protocols changed resulting in a decrease of morbidity and mortality over the past 2 decades. Here, transfusion and fluid resuscitation practices were analysed in severe injured children in Germany.

**Methods:**

Severely injured children (maximum Abbreviated Injury Scale (AIS) ≥ 3) admitted to a certified trauma-centre (TraumaZentrum DGU®) between 2002 and 2017 and registered at the TraumaRegister DGU® were included and assessed regarding blood transfusion rates and fluid therapy.

**Results:**

5,118 children (aged 1–15 years) with a mean ISS 22 were analysed. Blood transfusion rates administered until ICU admission decreased from 18% (2002–2005) to 7% (2014–2017). Children who are transfused are increasingly seriously injured. ISS has increased for transfused children aged 1–15 years (2002–2005: mean 27.7–34.4 in 2014–2017). ISS in non-transfused children has decreased in children aged 1–15 years (2002–2005: mean 19.6 to mean 17.6 in 2014–2017). Mean prehospital fluid administration decreased from 980 to 549 ml without affecting hemodynamic instability.

**Conclusion:**

Blood transfusion rates and amount of fluid resuscitation decreased in severe injured children over a 16-year period in Germany. Restrictive blood transfusion and fluid management has become common practice in severe injured children. A prehospital restrictive fluid management strategy in severely injured children is not associated with a worsened hemodynamic state, abnormal coagulation or base excess but leads to higher hemoglobin levels.

## Introduction

Trauma is the leading cause of death in children [1], with severe haemorrhage as the primary contributing factor [2–4]. Massive transfusion in children is known to be associated with several risks resulting in significant morbidity and mortality (3). Treatment of those severely injured children is a challenge due to differences in physiology and anatomy. Fluid and transfusion management plays an important role in resuscitation of severe haemorrhage not only in children.

While blood transfusion rates in serious injured adults decreased over the last decade and were associated with a reduction in morbidity and mortality [5], there are no sufficient data that confirm a similar change in practice in children. Similarly, positive fluid balance has been associated with higher mortality rates in adult trauma patients (5), but no data for fluid management in paediatric trauma patients are available. We hypothesized, that similar to adults, seriously injured children also receive less blood transfusion and less fluids. This hypothesis was tested by an analysis of the TraumaRegister DGU® (TR-DGU).

## Materials and methods

The TraumaRegister DGU® (TR-DGU) of the German Trauma Society (DGU) was established in 1993. The aim of this multi-centre database is a pseudonymised and standardised documentation of severely injured patients [6].

Data are collected prospectively for four consecutive time periods from the site of the accident until discharge from hospital: (a) pre-hospital phase, (b) emergency room and initial surgery, (c) intensive care unit and (d) hospital discharge. The documentation includes detailed information on patient demographics, injury pattern, comorbidities, pre- and in-hospital management, course on intensive care unit, relevant laboratory findings including data on transfusion and outcome of each individual. The inclusion criteria are admission to hospital via emergency room with subsequent intensive care unit (ICU) / intensive care medicine (ICM) care or admission to the hospital with vital signs and death before admission to ICU.

The infrastructure for documentation, data management, and data analysis is provided by the “AUC—Academy for Trauma Surgery”, a company affiliated to the DGU. The scientific leadership is provided by the “Committee on Emergency Medicine, Intensive Care and Trauma Management (Sektion NIS)” of the DGU. The participating hospitals submit their data pseudonymised into a central database via a web-based application. Scientific data analysis is approved according to a peer review procedure, as provided by the publication’s guideline of TR-DGU. The participating hospitals are primarily located in Germany (90%) but a rising number of hospitals of other countries contribute data as well (at the moment from Austria, Belgium, China, Finland, Luxembourg, Slovenia, Switzerland, The Netherlands, and the United Arab Emirates). Currently, approximately 33,000 cases from more than 650 hospitals are registered into the database annually.

Participation in TR-DGU is voluntary. However, for hospitals associated with TraumaNetzwerk DGU®, the entry of at least a basic data set is mandatory as part of the quality management program.

The present study has been reviewed by the TR-DGU review board and is registered under the TR-DGU project id: 2019–049. The study was performed in accordance with the Declaration of Helsinki.

### Inclusion criteria

**Fig. 1:**
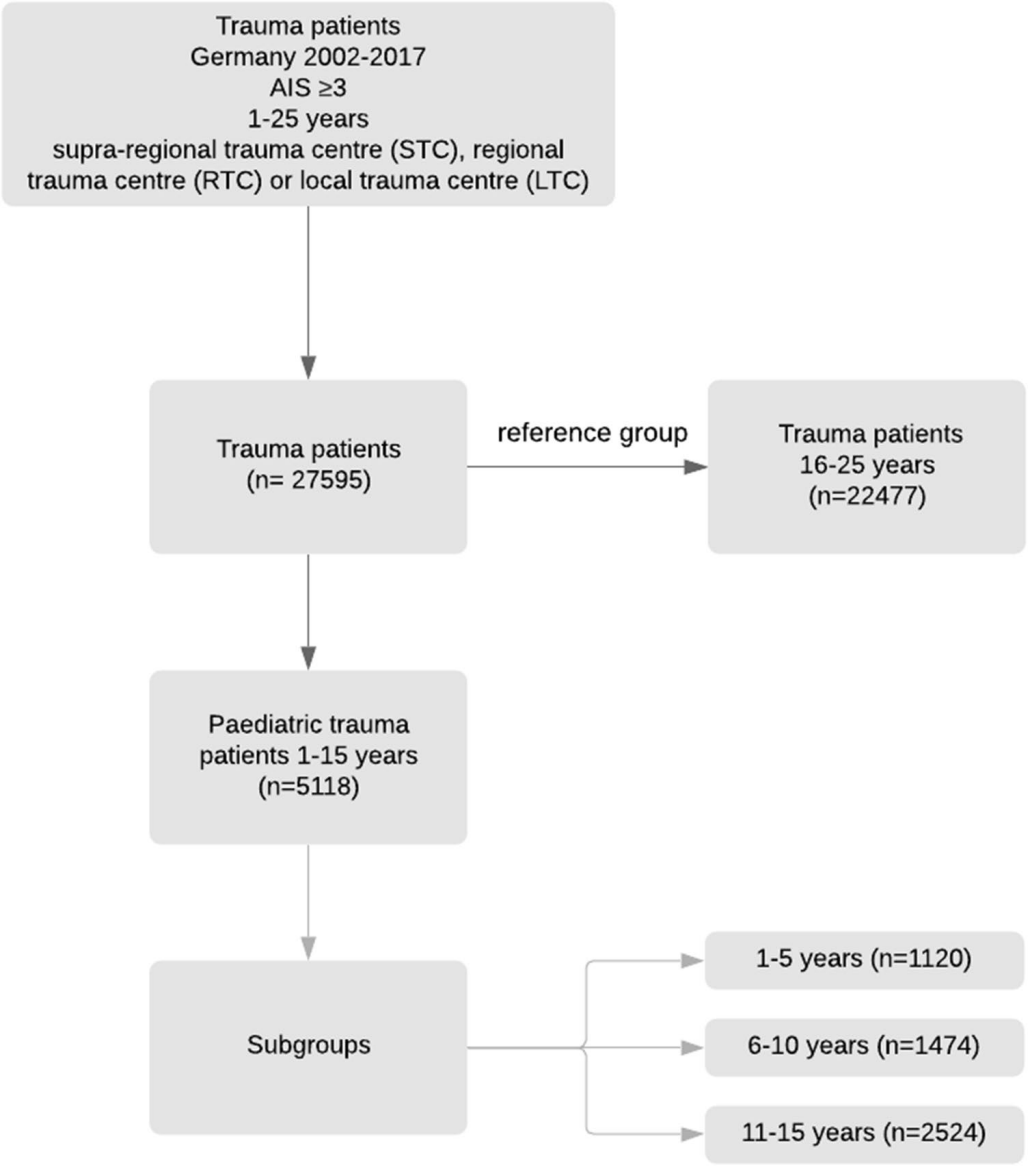
Flow sheet patient inclusion. Patients (aged 1–15 years) for primary analysis and young adults (16–25 years) as reference were included with an Abbreviated Injury Scale (AIS) of 3 or higher who were directly admitted to a German trauma centre (TraumaZentrum DGU®) from 2002 to 2017

Patients (aged 1–15 years) for primary analysis and young adults (16–25 years) as reference were included with an Abbreviated Injury Scale (AIS) of 3 or higher (serious injury) [7] who were directly admitted to a German trauma centre (TraumaZentrum DGU®) from 2002 to 2017. Early transfers out (< 48 h after admission) were excluded due to missing final outcome. Children younger than 1 year of age were excluded due to limited sample sizes (median *n* = 11/year). Paediatric patient groups were defined by 1–15 as well as subgroups of 1–5, 6–10 and 11–15 years of age (Fig. 1).

### Statistical analysis

Statistical analysis was performed using IBM® SPSS® Statistics (Version 24, IBM®, Armonk, New York). Univariable and multivariable analysis were performed. Descriptive results are presented as count/ percentages, median or mean ± standard deviation (SD). Multivariable analysis based on a logistic regression model was performed for risk of death analysis: RISC II score was combined with blood transfusion [8]. A *p* value of < 0.05 was considered statistically significant.

Injury Severity Score (ISS), systolic blood pressure on scene, haemoglobin-concentration and base excess were compared. Mortality between patients received transfusion and those who did not has been analysed.

## Results

A total of 27,595 serious injured patients aged 1–25 including 5118 children (aged 1–15 years) were identified between 2002 to 2017 (Fig. 1). While the median ISS of young adult (16–25 years) and older children (11–15 years) dropped over the years (2002–2017) from 22 to 18 and from 19 to 17, the ISS for children 1–10 (median 16) did not change significantly. A decrease of the ISS is also shown in the subgroup analysis by age and years (Table 1). More boys were seriously injured than girls (Table 2). In children older than 5 years of age and young adults, road traffic collisions were the leading cause for hospital admission. Leading injury in children aged 1–5 years is severe brain injury (Table 2).

**Table 1:** Development of the Injury Severity Score by years and age groups

ISS	1–5	6–10	11–15	16–25
2002–2005 (mean, SD)	19.8 ± 13.0	19.4 ± 11.6	22.5 ± 12.7	24.7 ± 13.7
2006–2009 (mean, SD)	21.5 ± 13.3	19.5 ± 10.8	23.3 ± 13.0	25.6 ± 13.8
2010–2013 (mean, SD)	20.4 ± 13.7	18.2 ± 11.0	20.6 ± 11.7	22.4 ± 12.8
2014–2017 (mean, SD)	19.3 ± 12.6	18.0 ± 10.3	19.2 ± 10.8	21.0 ± 12.1

**Table 2:** Patient characteristics

Age	1–5 (*n* = 1120)	6–10 (*n* = 1474)	11–15 (*n* = 2524)	16–25 (*n* = 22,477)
Sex male (%, *n*)	62.5% (698)	64.8% (954)	63.3% (1595)	75.1% (16,836)
Sex female (%, *n*)	37.5% (419)	35.2% (519)	36.7% (926)	24.9% (5585)
Mean ISS (SD)	20.0 (13.1)	18.4 (10.7)	20.6 (11.7)	22.5 (12.9)
Transfusion until ICU admission (%, *n*)	11.4% (128)	6.1% (90)	10.0% (252)	15.1% (3388)
Additional FFP, if transfused (%, *n*)	59.4% (41)	62.5% (60)	50.0% (75)	45.2% (70)
Traffic accident (%, *n*)	39.5% (429)	64.2% (927)	66.7% (1657)	75.6% (16,722)
Transport by rescue helicopter (%, *n*)	35.6% (377)	35.5% (508)	31.6% (762)	29.9% (6481)
Intubation (%,* n*)	38.4% (411)	35.7% (511)	38.3% (942)	41.0% (9084)
Severe brain injury (%, *n*)	62.2% (697)	52.9% (780)	47.2% (1192)	39.1% (8778)

*ISS* Injury Severity Score; *pRBC* packed red blood cells

### Blood transfusion

**Fig. 2:**
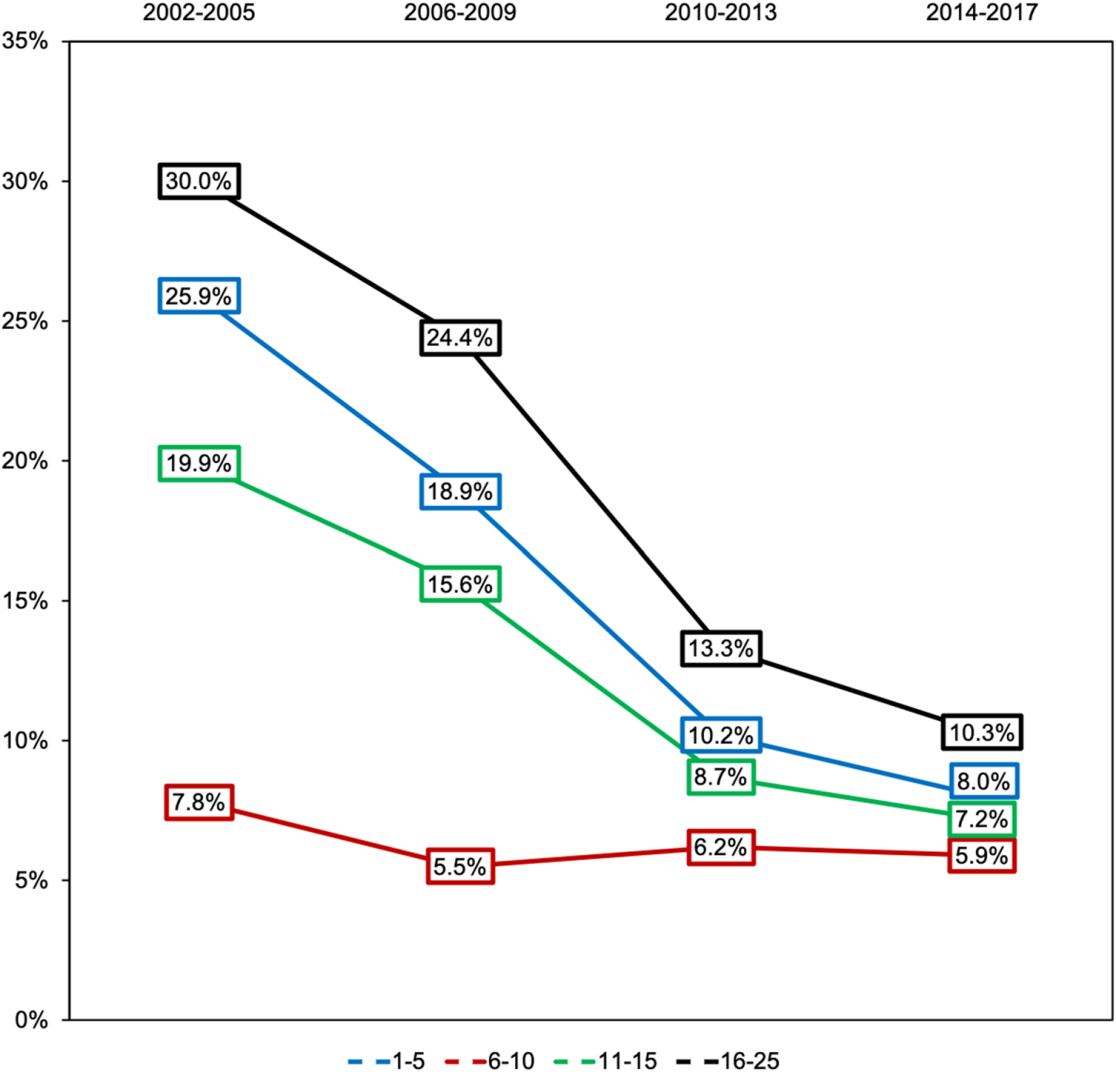
Development of transfusion rates. Development of transfusion rates in serious injured children and young adults 2002–2017

Blood transfusion rates (administered until ICU admission) decreased from 17.6% during the observation period of 2002–2005 to 7.0% in 2014–2017. Subgroup analysis showed a decrease in all age sub-groups: Blood transfusion decreased from 7.8% (2002–2005) to 5.9% (2014–2017; 6–10 years) and from 25.9% to 8.0% (1–5 years) (Fig. 2). In young adults (16–25 years) transfusion rates decreased also from 30.0% for the period of 2002–2005 to 10.3% in 2014–2017. In-hospital mortality rate for transfused children (1–15) increased from 21.7% (2002–2005) to 41.9% (2014–2017).

Multivariable risk of death analysis (RISC II Score + transfusion) showed a higher in-hospital mortality in transfused children than in not transfused children (OR = 1.61, 95% Confidence interval 1.10–2.35). Mortality rates in transfused children were higher than predicted by RISC II scores (Table 3). ISS has risen in transfused children aged 1–15 years (2002–2005: mean 27.7 to mean 34.4 in 2014–2017). The ISS for non-transfused children fell in children aged 1–15 years (2002–2005: mean 19.6 to mean 17.6 in 2014–2017) (Table 3).

**Table 3:** Mortality rates with and without blood transfusion in 1–15-year-old children

Year	*n*	Mortality overall (%)	Mortality if not transfused (%)	Mortality if transfused (%)	ISS if not transfused (Mean, SD)	ISS if transfused (Mean, SD)	RISC II prognosis if transfused (%)
2002–2005	393	9.7	7.1	21.7	19.6 (12.0)	27.7 (12.9)	19.4
2006–2009	702	8.1	5.0	28.1	20.0 (10.8)	34.4 (15.6)	29.5
2010–2013	1811	7.7	5.2	36.0	18.4 (10.2)	36.3 (16.9)	36.5
2014–2017	2212	6.6	3.9	41.9	17.7 (9.6)	34.4 (16.3)	36.7

*pRBC* packed red blood cells

### Fluid resuscitation, coagulation and blood pressure

**Fig. 3:**
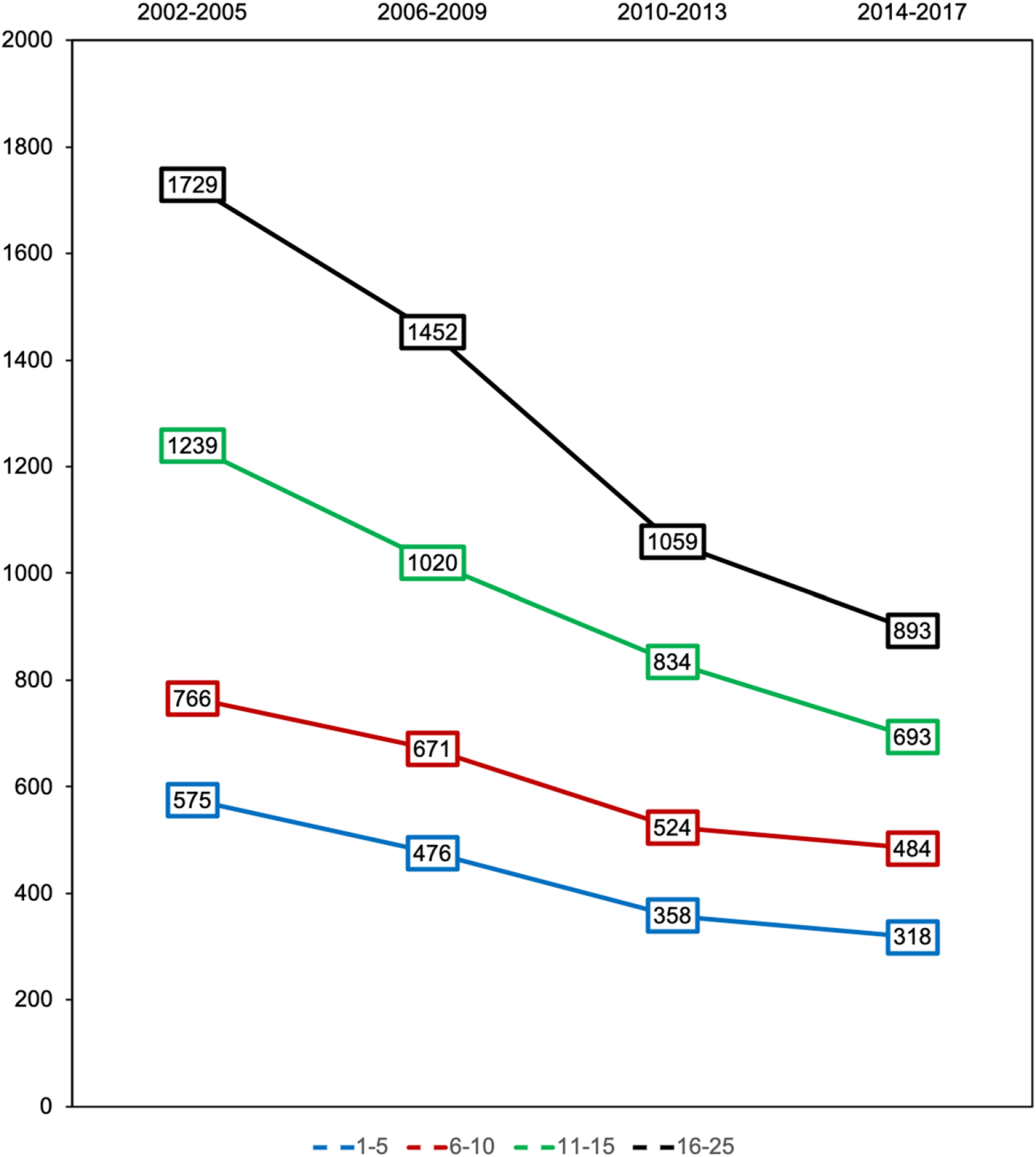
Development of prehospital fluid therapy. Development of mean prehospital fluid therapy (ml) in serious injured children and young adults 2002–2017

The amount of administered prehospital intravenous fluid decreased in all age groups (Fig. 3). While 1–15-year-old children received in mean 980 ml of fluids in the years 2002–2005, only 549 ml (mean) were administered between 2014–2017. Fluid resuscitation in the Emergency Room (ER) decreased from mean 1685 ml to mean 765 ml (Tables 4 and 5) during the respective periods. First Base Excess measured in ER increased from mean -3.41 to 2.34 mmol/l between the 2 observed periods. Mean Hemoglobin Concentration 10.96 and 12.0 g/dl, respectively, and mean International Normalized Ratio (INR) 1.31 and 1.2, respectively, were not significantly affected. Documented prehospital and first ER systolic blood pressure increased over the years (Table 5).

**Table 4:** Fluid therapy, coagulation and blood pressure in ER 1–15 years

Years	RR Syst. PH	RR Syt. ER	Hb Level ER	INR ER	BE ER	Fluids PH	Fluids ER
	mmHG	mmHG	g/dl		mmol/l	ml	ml
2002–2005 (mean, SD)	106 (25)	113 (22)	11.0 (2.2)	1.31 (0.47)	– 3.4 (4.6)	980 (779)	1685 (1656)
2006–2009 (mean, SD)	109 (24)	116 (24)	11.3 (2.2)	1.30 (0.68)	– 3.1 (5.5)	829 (663)	1313 (1260)
2010–2013 (mean, SD)	109 (28)	112 (26)	11.9 (2.1)	1.22 (0.46)	– 2.8 (5.0)	649 (554)	745 (852)
2014–2017 (mean, SD)	114 (28)	116 (25)	12.0 (2.0)	1.20 (0.53)	– 2.3 (4.8)	549 (450)	766 (982)

*PH* prehospital; *ER* emergency room; *INR* International Normalized Ratio; *BE* base excess

**Table 5:** INR, BE, RR systolic in ER

INR	1–5	6–10	11–15	16–25
2002–2005 (mean, SD)	1.30 ± 0.39	1.30 ± 0.38	1.32 ± 0.54	1.37 ± 084
2006–2009 (mean, SD)	1.38 ± 0.87	1.21 ± 0.49	1.32 ± 0.67	1.35 ± 0.82
2010–2013 (mean, SD)	1.29 ± 0.72	1.22 ± 0.47	1.20 ± 0.30	1.24 ± 0.64
2014–2017 (mean, SD)	1.28 ± 0.75	1.20 ± 0.55	1.19 ± 0.39	1.19 ± 0.55

BE	1–5	6–10	11–15	16–25
2002–2005 (mean, SD)	− 4.1 ± 4.7	− 3.2 ± 3.6	− 3.3 ± 5.1	− 3.5 ± 4.7
2006–2009 (mean, SD)	− 5.0 ± 6.0	− 3.8 ± 3.8	− 2.2 ± 5.9	− 3.3 ± 4.7
2010–2013 (mean, SD)	− 4.4 ± 5.9	− 2.8 ± 5.2	− 2.1 ± 4.4	− 2.6 ± 4.8
2014–2017 (mean, SD)	− 3.8 ± 5.7	− 2.1 ± 4.4	− 1.8 ± 4.4	− 2.0 ± 4.7

RR Sys. prehospital	1–5	6–10	11–15	16–25
2002–2005 (mean, SD)	106 ± 20	103 ± 31	108 ± 26	113 ± 29
2006–2009 (mean, SD)	101 ± 21	107 ± 27	113 ± 26	114 ± 28
2010–2013 (mean, SD)	100 ± 31	106 ± 26	114 ± 27	118 ± 29
2014–2017 (mean, SD)	102 ± 34	112 ± 25	119 ± 26	123 ± 29

*INR* International Normalized Ratio; *BE* base excess; *SD* standard deviation

## Discussion

Blood transfusion and fluid resuscitation practices changed over a 16-year period in Germany. Resuscitation and treatment of severely injured children is a significant challenge for all professionals involved. Children are a physiologically inhomogeneous group ranging from neonates to adolescents. Children who have suffered severe hemorrhage or have ongoing bleeding may require life-saving blood transfusion, however, an underestimation of blood loss is common [9].

We were able to demonstrate in our study that severely injured children receive fewer transfusions. The reasons for this cannot be directly deduced from the presented study. It remains to be assumed that more careful use of blood products, as it has been observed in the therapy of adults, has also been adopted in the care of children [5]. The reduction of fluid administration leads to a higher Hb level, which we were also able to show, and thus probably to fewer transfusion indications. At the same time, the coagulation system is less affected by fewer fluids being applied, which can lead to reduced blood loss.

Blood transfusions are recommended for hemoglobin < 5 g/dl in non-life-threatening bleeding [1, 10]. However, there is no evidence or clear guidance for children with haemoglobin between 5 and 7 g/dl. The European Society of Anaesthesiology recommends transfusion targets for bleeding children 7–9 g/dl [11]. In paediatric cardiac surgery, no differences in mortality, clinical outcome or complication between restrictive (hemoglobin 8.0 g/dl) and liberal group (10.8 g/dl) have been found [12]. In subgroup of children with severe traumatic brain injury hemoglobin concentrations between 7–10 g/dl are recommended [13]. There is little evidence regarding transfusion triggers, but rather expert opinions [9], as a recent review article also points out [14]. However, the validity of haemoglobin concentration should also be considered in bleeding children. Measured hemoglobin concentrations are only reliably associated with actual blood loss after adequate fluid replacement. Haemoglobin concentrations may approximately be normal within the first hour of therapy, if no adequate fluid resuscitation was provided. We recommend measuring Hb, but with normal Hb levels and suspected relevant blood loss at the same time, the result should be critically reviewed to determine whether sufficient fluids have been substituted. However, an individual indication for blood transfusion should be considered and include setting, pre-existing disease, injury and parameters for insufficient oxygen delivery such as lactatic acidosis.

The use of massive transfusion protocols in pediatric trauma patients has not led to an improvement of in-hospital mortality or outcomes [15]. However, we still recommend preparation for challenging medical situations with supporting aids such as treatment protocols and dosage recommendations. It can be assumed that the recommendations are beneficial for faster and safer care [16]. In hemorrhagic shock a blood product ratio of 2:1:1 to 1:1:1 (RBCs, plasma, platelets) is recommended [10].

Although no data for the use of vasoactive drugs were available for analysis, it must be assumed that these were frequently used as lower rates of transfusion and infusion were described without a decline in systolic blood pressure measurements. One explanation for this may be that more expertise on the use of catecholamines has been developed in the therapy of children. The reason for this could be that pediatric emergency rulers or apps are now part of the standard equipment of emergency medical services and support the emergency physician in calculating the catecholamine dose [16]. Moreover, the use of pelvic slings and tourniquets, which are now widely available to reduce acute blood loss, leads to less blood loss and thus to higher blood pressure upon arrival in the emergency room. Patient Blood Management (PBM) addresses evidence-based transfusion medicine practice and is recommended by the World Health Organization (WHO) in order to avoid transfusion associated risks such as infections, anaphylaxis, transfusion-associated graft-versus-host diseases or immunomodulation [17]. Implementing principles to optimize erythropoiesis, minimizing blood loss and managing anaemia leading to minimization of allogenic blood transfusion are essences of this program [18, 19]. Giving attention to the point that incidence of adverse outcome in transfused pediatric patients is 1.3 times higher in children and 2.8 higher in neonates when compared with adults [20], implementing PBM programs in paediatric settings is a relevant and current topic [21–24]. Several strategies already implemented in adult PBM Programs can easily be implemented in paediatric care. In context of initial management of severe bleeding avoiding hypothermia, acidosis, low calcium concentration [19] and administration of tranexamic acid are recommended and should be part of an paediatric massive haemorrhage protocol. The implementation of localised PBM programs and recommendations on RBC transfusion thresholds may have led to a reduction of transfusion rates in paediatric trauma patients as shown.

The observed increase in mortality in transfused pediatric trauma patients is most likely due to selection bias, as these transfused children also show increased ISS over time. A similar trend of an increased mortality of transfused patients following a stricter transfusion policy was also described in adult trauma patients [5]. RISC II prognosis score makes its prognosis based on the findings at the time of admission to the emergency room. The fact that transfusions were subsequently required is an indication of a possible deterioration or complication (e.g., bleeding could not be stopped). This may further explain the difference in observed versus predicted mortality. It must also be considered that the participating hospitals in the registry have changed over the years. In the beginning, only level 1 hospitals (hospitals of maximum care) sent data, only from 2012/2013 it´s safe to assume that all trauma in Germany are included in the analysis.

Reduction of prehospital admitted fluid resulted in higher hemoglobin concentrations in ER in this study. This is consistent with findings by Hussman et al. that demonstrate an association of increased volume replacement and higher blood transfusion rates, coagulopathy and increased mortality [25]. Aggressive fluid resuscitation is also known to be an independent risk factor after trauma [26] and is associated with fluid-related complications [27]. Comparable paediatric trauma data are available from Defence Trauma Registry of the US military for children suffering from blast injuries [28]. Normalization of INR, BE and systolic blood pressure under reduced fluid replacement have been observed in adults as well as in this current study [26].

Acute traumatic coagulopathy (ATC) is an early-onset syndrome and is common in adult and paediatric trauma patients [29, 30]. ATC is an independent predictor for mortality in children after severe trauma [31]. The TraumaRegister DGU^®^ registers INR at admission to the ER as a standard parameter for coagulopathy. INR was reduced in the last quarter of our analysis, but coagulopathy as defined as an INR > 1.2 is still common overall. Real time measurement of coagulation might be useful in treating severe injured children. Standard coagulation tests do not necessarily correlate with coagulopathies such as Shutdown Fibrinolysis and Acute Traumatic Coagulopathy (ATC). Viscoelastic haemostatic results are available only in a few cases of severe injured children in The TraumaRegister DGU®. Viscoelastic haemostatic assays (VHA) are currently not standard of care in bleeding paediatric trauma patients but may provide valuable data for goal-directed haemostatic therapy in these patients [32, 33]. Prospective studies investigating viscoelastic monitoring-based therapy in paediatric trauma patients are needed.

## Limitations

The data are taken from the TraumaRegister DGU®. Due to a possible selection error, register data are less valid than data generated by prospective randomized studies, so that conclusions from the observed phenomena must be evaluated with the necessary carefulness.

## Conclusions

The current study provides evidence that restrictive blood transfusion and fluid management has become a common practice in severe injured children. A prehospital restrictive fluid management strategy in severely injured children is not associated with a worsened hemodynamic state, abnormal coagulation, or base excess but leads to higher hemoglobin levels. Further prospective studies are needed to investigate both transfusion and fluid concepts in paediatric trauma. The objective of such studies should be the implementation of treatment protocols for fluid and haemotherapy to provide recommendations based on a valid database.
